# Crystal structure and electrical properties of bismuth sodium titanate zirconate ceramics

**DOI:** 10.1186/1556-276X-7-57

**Published:** 2012-01-05

**Authors:** Ampika Rachakom, Panupong Jaiban, Sukanda Jiansirisomboon, Anucha Watcharapasorn

**Affiliations:** 1 Department of Physics and Materials Science, Faculty of Science, Chiang Mai University, Chiang Mai, 50200, Thailand; 2 Materials Science Research Center, Faculty of Science, Chiang Mai University, Chiang Mai, 50200, Thailand

**Keywords:** ceramics, X-ray diffraction, dielectric properties, ferroelectricity

## Abstract

Lead-free bismuth sodium titanate zirconate (Bi_0.5_Na_0.5_Ti_1-x_Zr_x_O_3 _where *x *= 0.20, 0.35, 0.40, 0.45, 0.60, and 0.80 mole fraction) [BNTZ] ceramics were successfully prepared using the conventional mixed-oxide method. The samples were sintered for 2 h at temperatures lower than 1,000°C. The density of the BNTZ samples was at least 95% of the theoretical values. The scanning electron microscopy micrographs showed that small grains were embedded between large grains, causing a relatively wide grain size distribution. The density and grain size increased with increasing Zr concentration. A peak shift in X-ray diffraction patterns as well as the disappearance of several hkl reflections indicated some significant crystal-structure changes in these materials. Preliminary crystal-structure analysis indicated the existence of phase transition from a rhombohedral to an orthorhombic structure. The dielectric and ferroelectric properties were also found to correlate well with the observed phase transition.

## Background

Lead-based PbTiO_3_-PbZrO_3 _solid solutions have dominated the market of actuator and sensor materials due to their excellent ferroelectric and piezoelectric properties. In particular, a compositional ratio of Zr/Ti of around 52/48 showed the morphotropic phase boundary between a tetragonal and a rhombohedral phase, where enhanced polarizability and optimum domain orientation were observed [1-6]. However, PbO loss during high-temperature processes is considered to be environmental pollution with additional problems of recycling and waste disposal. Therefore, researchers have attempted to develop new lead-free smart materials in order to replace the lead-based ones [7]. BaTiO_3 _is one example of the most commonly used lead-free material for capacitors and actuators due to its inherent ferroelectric nature. However, its main disadvantage is the narrow working temperature; therefore, the use of a BaTiO_3_-BaZrO_3 _solid solution with the addition of Zr up to 30% mole was investigated [8-10]. The materials were found to exhibit a composition-induced phase transition from normal to relaxor ferroelectric with a higher dielectric constant than both PZT and BaTiO_3_. This allowed the materials to be used over a broader temperature range. Following these studies, this paper was aimed to study Bi_0.5_Na_0.5_TiO_3_-Bi_0.5_Na_0.5_ZrO_3 _solid solutions with the addition of a Zr concentration from 0.20, 0.35, 0.40, 0.45, 0.60, and 0.80 mole fraction. The relationship between the phase, crystal structure, and electrical properties is investigated and discussed.

## Methods

Bi_0.5_Na_0.5_Ti_1-x_Zr_x_O_3 _compositions were prepared using the mixed-oxide method incorporating Bi_2_O_3 _(> 98%, Fluka, Sigma-Aldrich Corporation, St. Louis, MO, USA), Na_2_CO_3 _(99.5%, Carlo Erba Reagenti SpA, Rodano, Italy), TiO_2 _(> 99%, Riedel de Haën, Sigma-Aldrich Corporation, St. Louis, MO, USA), and ZrO_2 _(> 99%, Riedel de Haën) in stoichiometric proportions. The mixed powders were ball milled in ethanol for 24 h using zirconia milling media and calcined at 800°C for 2 h. The calcined Bi_0.5_Na_0.5_Ti_1-x_Zr_x_O_3 _powders were then ball milled again for 6 h and uniaxially pressed at a pressure of 5.5 MPa with a few drops of 3 wt.% polyvinyl alcohol to bind it into disks of 10-mm diameter and 1- to 1.5-mm thickness. The disks were the sintered at 900°C for 2 h, except for the sample with 0.20 mole fraction Zr which was sintered at 950°C for 2 h, in air. The X-ray diffractometer (Philip Model X-pert, PANalytical B.V., Almelo, The Netherlands) with CuKα radiation was used to investigate the phase and crystal structure of the sintered ceramics. The preliminary crystal structure details were calculated using the Powder Cell program [11], which is based on the X-ray diffraction pattern of lead-free bismuth sodium titanate zirconate (Bi_0.5_Na_0.5_Ti_1-x_Zr_x_O_3 _where *x *= 0.20, 0.35, 0.40, 0.45, 0.60, and 0.80 mole fraction) [BNTZ] ceramics. The bulk densities of the sintered ceramics were measured using Archimedes' method. The theoretical density was approximated from the unit cell size and its constituent ions. Scanning electron microscopy [SEM] (JEOL JSM-6335F, JEOL Ltd., Akishima, Tokyo, Japan) was used to observe the microstructure of the ceramics. To prepare the SEM samples, they were well-polished and thermally etched for 15 min at 750°C. The average grain size was then evaluated from these SEM images. The room temperature dielectric constant [*ε*_r_] and dielectric loss [tan *δ*] were measured with an LCR meter (LF Impedance Analyzer 4292A, Agilent Technologies Inc., Santa Clara, CA, USA), but the ferroelectric hysteresis loops were measured in a silicone oil bath using a modified Sawyer-Tower circuit.

## Results and discussion

**Figure 1:**
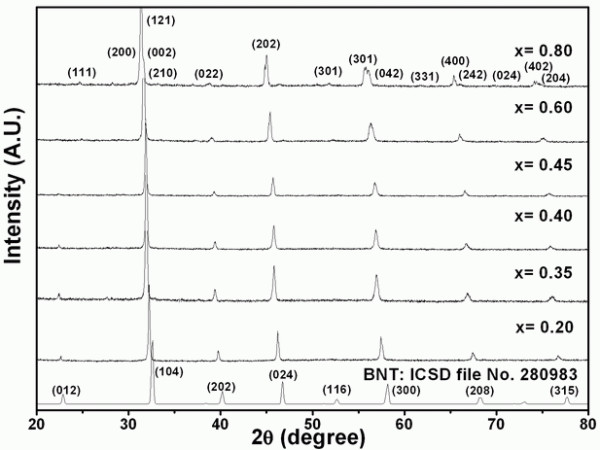
**X-ray diffraction patterns of Bi_0.5_Na_0.5_Ti_1-x_Zr_x_O_3 _ceramics**. Where *x *= 0.20, 0.35, 0.40, 0.45, 0.60, and 0.80 mole fraction.

**Figure 2:**
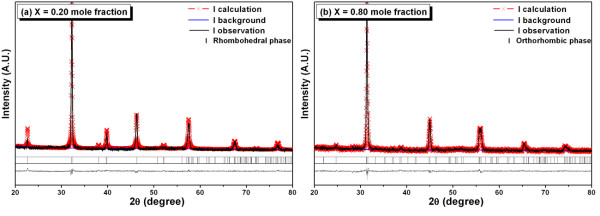
**Refinement of Bi_0.5_Na_0.5_Ti_1-x_Zr_x_O_3 _ceramics**. The refinement at (**a**) 0.20 mole fraction and (**b**) 0.80 mole fraction showed a rhombohedral phase and an orthorhombic phase, respectively.

X-ray diffraction patterns of Bi_0.5_Na_0.5_Ti_1-x_Zr_x_O_3 _ceramics where *x *= 0.20, 0.35, 0.40, 0.45, 0.60, and 0.80 mole fraction are shown in Figure 1. The BNTZ phase could be matched with pure BNT (ICSD file no. 280983) for the rhombohedral space group R3c [12,13]. With the presence of Zr, all reflection peaks systematically shifted to angles lower than 2$\underset{\cdot }{\theta }$. This observation suggested that the Zr^4+^-ion substitution into the Ti^4+ ^site led to an enlargement of the unit cell [9,10] which corresponded to the fact that the ionic radius of Zr^4+ ^(*r*_Zr_^4+ ^= 0.72 Å [14]) was larger than that of Ti^4+ ^(*r*_Ti_^4+ ^= 0.605 Å [14]). Accompanying the shift, intensities of some diffraction peaks such as (012) and (202) were reduced, indicating that lattice distortion alongside unit cell expansion has occurred. The refinement of the X-ray diffraction patterns was carried out, and the results are listed in Table 1. The refined patterns for the Zr compositions equal to 0.2 and 0.8 are also shown in Figure 2. From these data, BNTZ ceramics containing Zr from 0.2 to 0.6 possessed a rhombohedral structure with increased lattice parameters. The increase in the value of the interaxial angle caused the structure to be close to cubic, which resulted in the disappearance of certain reflections. For Zr = 0.8, Figures 1 and 2 showed an apparent splitting of the (104) and (300) peaks in the original rhombohedral structure. Based on refinement results, the structure was orthorhombic having the lattice parameters shown in Table 1. This finding was somewhat in partial agreement with the orthorhombic structure previously obtained for Bi_0.5_Na_0.5_ZrO_3 _[15]. Hence, for this BNTZ solid solution ceramic system, the structure changed from rhombohedral to orthorhombic when the Zr concentration exceeded 0.6 mole fraction. The exact phase-transition composition is currently being investigated.

**Table 1:** Relationships between crystal structure and electrical properties of BNTZ ceramics

Bi_0.5_Na_0.5_Ti_1-x_Zr_x_O_3_ (mole fraction)	Lattice parameter/distortions		Relative density	Dielectric constant (*ε*_r_) at 100 kHz	**tan **$\underset{\cdot }{\delta }$
	*a*, *b*, *c *(Å)	α (°)			
0.20	3.9222	89.8600	94.7	445.8105	0.0878
0.35	3.9556	89.8675	97.7	453.3421	0.0811
0.40	3.9602	89.8713	96.8	320.9603	0.0706
0.45	3.9721	89.8719	96.1	313.1384	0.0627
0.60	3.9879	89.9247	96.8	239.9664	0.0668
0.80	*a *= 5.9663 *b *= 8.0883 *c *= 5.6664	90.000	97.9	196.2317	0.0439

**Figure 3:**
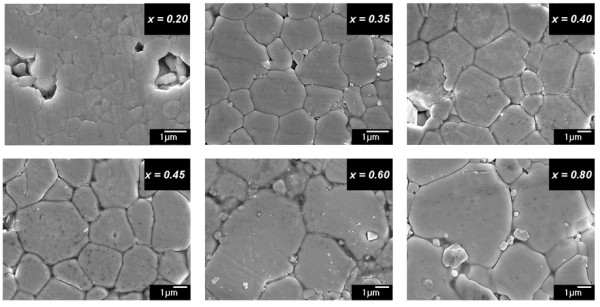
**SEM image of Bi_0.5_Na_0.5_Ti_1-x_Zr_x_O_3 _ceramics**. Where *x *= 0.20, 0.35, 0.40, 0.45, 0.60, and 0.80 mole fraction.

All BNTZ ceramics had experimental density values in the range of 5.8 to 6.1 g/cm^3 ^as shown in Table 1 which corresponded to the relative densities of around 95% of the theoretical densities. For the 0.20 mole fraction of Zr, the sample was sintered at 950°C for 2 h due to the influence of a high Ti concentration [16,17]. As the amount of Zr increased, the sintering temperature could be lowered to 900°C. This seemed to be a typical behavior of solid solutions whose melting points might be lowered by adding Zr as a deducted form of the lattice expansion. The difference in sintering behavior could also be observed from the microstructure of BNTZ ceramics; all samples were dense with well-defined grains (Figure 3). The ceramic containing Zr = 0.2 possessed an average grain size of about 0.8 μm, whereas the presence of Zr ions generally caused the grain size to increase. The enhanced ability for ionic diffusion in BNTZ ceramics seemed to support the possible lowering of the melting point of these solid solutions.

The *ε*_r _and tan $\underset{\cdot }{\delta }$ of Bi_0.5_Na_0.5_Ti_1-x_Zr_x_O_3 _ceramics, at the frequency of 100 kHz, are tabulated in Table 1. In general, increasing Zr concentration in BNTZ ceramics caused a gradual decrease in dielectric constant with a slight decrease in dielectric loss. This behavior was in agreement with other systems with isovalent additives [2]. In addition, the replacement of larger Zr ions may also cause the dipoles to be poorly induced due to limited ionic movement. This decreasing trend was observed through the sample with a composition of Zr = 0.8, whose structure was orthorhombic. It seemed that the effect of ionic size and limited ionic movement in the perovskite structure of this compound had a greater influence on the dielectric properties than the change in the crystal structure in their unit-all dimentions.

**Figure 4:**
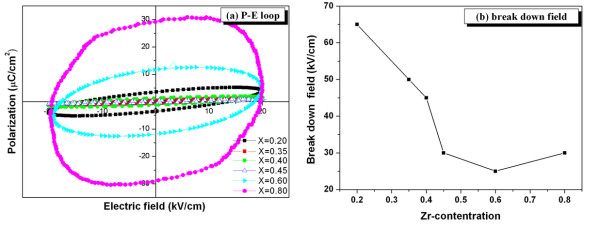
**P-E hysteresis loops (a) and the breakdown field (b) of Bi_0.5_Na_0.5_Ti_1-x_Zr_x_O_3 _ceramics**. Where *x *= 0.20, 0.35, 0.40, 0.45, 0.60, and 0.80 mole fraction.

Figure 4a, b illustrates the polarization-electric field [P-E] hysteresis loops and the breakdown field strengths of BNTZ ceramics, respectively. The hysteresis loops were obtained at the maximum applied electric field of 20 kV/cm and a frequency of 50 Hz. The shape of the P-E loops varied greatly with the ceramic composition. Up to Zr = 0.45 mole fraction, the loops showed an ellipse shape due to the vertical deflection electric field with partial dielectric displacement and partly due to conduction [1]. Limited domain reorientation might also be the cause of poor hysteresis loops for these compositions. For samples with Zr = 0.6 and 0.8, the loops showed higher values of remanent polarization though they were still unsaturated. This seemed to show the approximate transition point between the rhombohedral and orthorhombic structures. This was supported by an increase in the breakdown field strength for the Zr = 0.8 composition, which was partly due to the effect of a different crystal structure in this series of materials. Hence, this study showed that the observed dielectric and ferroelectric properties of BNTZ ceramics largely depended on compositional and crystal structure changes. Optimization of these properties could be achieved by fine-tuning the composition for specific applications.

## Conclusions

Lead-free Bi_0.5_Na_0.5_Ti_1-x_Zr_x_O_3 _(where *x *= 0.20, 0.35, 0.40, 0.45, 0.60, and 0.80 mole fraction) ceramics were successfully fabricated. X-ray diffraction patterns showed phase transition from rhombohedral to an orthorhombic structure. The addition of Zr concentration caused lattice expansion in agreement with ionic size consideration. All ceramic samples were dense with well-defined grain structures. The dielectric constant was found to decrease with increasing Zr content due to the larger-sized ionic substitution that limited dipole movement. Ferroelectric properties also showed compositional dependence due to the variation in domain reorientation ability. This study showed that electrical properties of BNTZ ceramics could be further improved by fine-tuning their composition for certain applications.

## Competing interests

The authors declare that they have no competing interests.

## Authors' contributions

AR carried out the bismuth sodium titanate zirconate experiment and analysis and drafted the manuscript. PJ participated as the assistant for the research experiment. AW and SJ participated in the conception and design of the study and revised the manuscript for important intellectual content. All authors read and approved the final manuscript.
